# Tetra­aqua­(1,10-phenanthroline-κ^2^ *N*,*N*′)cadmium(II) sulfate dihydrate

**DOI:** 10.1107/S1600536810028175

**Published:** 2010-07-21

**Authors:** Yuan-Yuan Zhang, Qiong-Hua Jin, Wei Yang, Cun-Lin Zhang

**Affiliations:** a Department of Chemistry, Capital Normal University, Beijing 100048, People’s Republic of China; b Beijing Key Laboratory for Terahertz Spectroscopy and Imaging, Key Laboratory of Terahertz Optoelectronics, Ministry of Education, Capital Normal University, Beijing 100048, People’s Republic of China

## Abstract

In the title mononuclear complex, [Cd(C_12_H_8_N_2_)(H_2_O)_4_]SO_4_·2H_2_O, the coordination geometry around the Cd^II^ atom is a distorted octa­hedron, with two aqua ligands occupying the axial positions. Inter­molecular O—H⋯O hydrogen bonds lead to the formation of a two-dimensional layer structure parallel to (001). The layers are connected by π–π inter­actions between the pyridyl and benzene rings of the phenanthroline ligands [centroid–centroid distances = 3.591 (1) and 3.610 (1) Å].

## Related literature

For general backgound to supra­molecular structures with coordination frameworks, see: Bie *et al.* (2006 ▶); Huang *et al.* (2010 ▶); Wu *et al.* (2009 ▶). For related structures, see: Li *et al.* (2003 ▶); Zheng & Lin (2003 ▶).
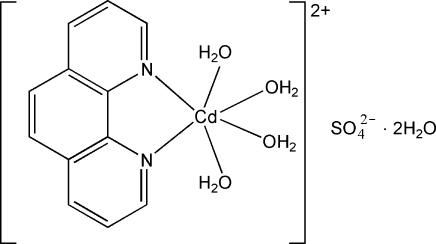


## Experimental

### 

#### Crystal data

[Cd(C_12_H_8_N_2_)(H_2_O)_4_]SO_4_·2H_2_O
*M* *_r_* = 496.78
Orthorhombic, 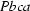

*a* = 8.8398 (9) Å
*b* = 18.6996 (19) Å
*c* = 22.349 (2) Å
*V* = 3694.3 (6) Å^3^
*Z* = 8
Mo *K*α radiation
μ = 1.35 mm^−1^
*T* = 298 K
0.41 × 0.30 × 0.27 mm

#### Data collection

Bruker APEX CCD diffractometer
Absorption correction: multi-scan (*SADABS*; Bruker, 2001 ▶) *T*_min_ = 0.622, *T*_max_ = 0.695
17188 measured reflections
3266 independent reflections
2789 reflections with *I* > 2σ(*I*)
*R*_int_ = 0.045

#### Refinement

*R*[*F* ^2^ > 2σ(*F* ^2^)] = 0.033
*wR*(*F* ^2^) = 0.065
*S* = 1.16
3266 reflections
236 parameters
H-atom parameters constrained
Δρ_max_ = 0.52 e Å^−3^
Δρ_min_ = −0.46 e Å^−3^

### 

Data collection: *SMART* (Bruker, 2007 ▶); cell refinement: *SAINT-Plus* (Bruker, 2007 ▶); data reduction: *SAINT-Plus*; program(s) used to solve structure: *SHELXS97* (Sheldrick, 2008 ▶); program(s) used to refine structure: *SHELXL97* (Sheldrick, 2008 ▶); molecular graphics: *SHELXTL* (Sheldrick, 2008 ▶); software used to prepare material for publication: *SHELXTL*.

## Supplementary Material

Crystal structure: contains datablocks global, I. DOI: 10.1107/S1600536810028175/hy2328sup1.cif

Structure factors: contains datablocks I. DOI: 10.1107/S1600536810028175/hy2328Isup2.hkl

Additional supplementary materials:  crystallographic information; 3D view; checkCIF report

## Figures and Tables

**Table 1:** Hydrogen-bond geometry (Å, °)

*D*—H⋯*A*	*D*—H	H⋯*A*	*D*⋯*A*	*D*—H⋯*A*
O1—H1*A*⋯O9^i^	0.85	1.85	2.682 (4)	167
O1—H1*B*⋯O4^ii^	0.85	2.10	2.952 (4)	176
O2—H2*A*⋯O5^iii^	0.85	1.83	2.682 (3)	178
O2—H2*B*⋯O8^iv^	0.85	1.93	2.757 (4)	165
O3—H3*A*⋯O8^iii^	0.85	1.95	2.787 (4)	170
O3—H3*B*⋯O7^ii^	0.85	1.90	2.746 (4)	170
O4—H4*A*⋯O7	0.85	1.84	2.679 (4)	171
O4—H4*B*⋯O6^iv^	0.85	1.87	2.686 (4)	159
O9—H9*A*⋯O5^iii^	0.85	2.00	2.844 (4)	172
O9—H9*B*⋯O10^iv^	0.85	1.92	2.768 (4)	176
O10—H10*A*⋯O8	0.85	2.05	2.875 (4)	162
O10—H10*B*⋯O6^v^	0.85	2.06	2.909 (4)	172

Symmetry codes: (i) 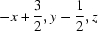; (ii) 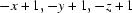; (iii) 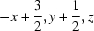; (iv) 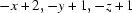; (v) 
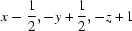
.

**Fig. 1.**
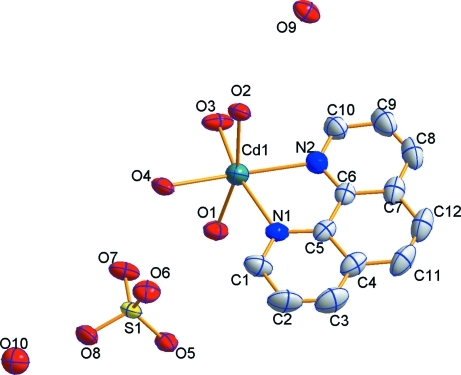
Molecular structure of the title complex. Displacement ellipsoids are drawn at the 50% probability level. H atoms are omitted for clarity.

**Fig. 2.**
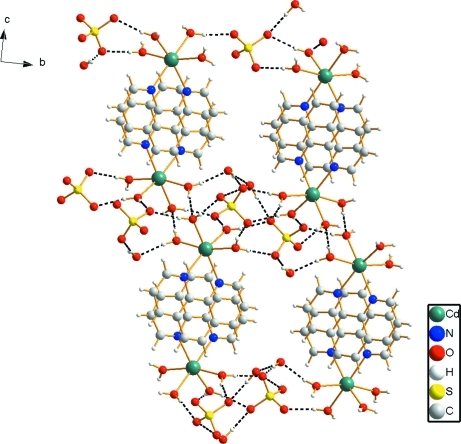
Crystal packing of the title complex. Dashed lines denote hydrogen bonds.

## supplementary crystallographic information

### Comment

Supramolecular compounds with coordination frameworks have received increasing attention due to their fascinating architectures and potential applications as functional materials of catalysis, magnetism, superconductor, non-linear optical materials and molecular recognition (Bie *et al.*, 2006; Huang *et al.*, 2010; Wu *et al.*, 2009). In this paper, we report the structure of the title compound, a new cadmium(II) complex.

The Cd^II^ atom is six-coordinated by two N atoms from a phenanthroline (phen) ligand, four O atoms from water molecules, forming a [Cd(phen)(H_2_O)_4_]^2+^ cation. The structure contains a sulfate anion to balance the charge and two uncoordinated molecules (Fig. 1). The complex cations, sulfate anions and water molecules are held together *via* O—H···O hydrogen bonds (Table 1), forming a two-dimensional layer. Interdigitation of phen ligands leads to the formation of a three-dimensional network (Fig.2), stabilized by significant π–π stacking interactions between the pyridyl and benzene rings of the phen ligands [centroid–centroid distances = 3.591 (1) and 3.610 (1) Å].

The Cd—O distances of 2.260 (3)–2.304 (2)Å in the title compound are slightly longer than the reported Cd—O distances of 2.246 (2)–2.325 (3) Å, and Cd—N distances of 2.317 (3)–2.337 (3)Å are shorter than the reported Cd—N distances of 2.318 (3)–2.351 (2)Å (Bie *et al.*, 2006). The cis O—Cd—O angles are in the range of 82.52 (9)–96.46 (11)° and the trans one is 165.28 (9)°. The cis N—Cd—O angles are in the range of 87.35 (10)–105.41 (10)° and the trans ones are 160.98 (12) and 168.48 (11)°. The N—Cd—N angle is 72.09 (11)°. These confirm the distorted octahedral environment around the Cd^II^ atom. It is noticable that in the similar complexes reported, [Cd(SO_4_)(C_12_H_8_N_2_)(H_2_O)_3_] (Li *et al.*, 2003), [Cd(C_12_H_8_N_2_)(H_2_O)_2_(SO_4_)]_n_ (Bie *et al.*, 2006) and [Mn(C_12_H_8_N_2_)(H_2_O)_2_(SO_4_)] (Zheng & Lin, 2003), the center metals are coordinated to O atom from sulfate, while in the title compound the sulfate acts as a free anion.

### Experimental

A mixture of CdSO_4_.8H_2_O (0.2 mmol), biimidazole (0.1 mmol), H_2_O (15 ml) and 1,10-phenanthroline (0.2 mmol) was sealed in a 25 ml Teflon-lined stainless-steel reactor and heated to 160°C for 72 h. After cooling, colorless crystals of the title compound were obtained.

### Refinement

C-bound H atoms were positioned geometrically and refined as riding atoms, with C—H = 0.93 Å and *U*_iso_(H) = 1.2*U*_eq_(C). The water H atoms were located in a difference Fourier map and refined as riding, with O—H = 0.85 Å and *U*_iso_(H) = 1.2*U*_eq_(O).

### Crystal data

**Table:**

[Cd(C_12_H_8_N_2_)(H_2_O)_4_]SO_4_·2H_2_O	*F*(000) = 2000.0
*M**_r_* = 496.78	*D*_x_ = 1.786 Mg m^−^^3^
Orthorhombic, *P**b**c**a*	Mo *K*α radiation, λ = 0.71073 Å
Hall symbol: -P 2ac 2ab	Cell parameters from 8439 reflections
*a* = 8.8398 (9) Å	θ = 2.2–28.1°
*b* = 18.6996 (19) Å	µ = 1.35 mm^−^^1^
*c* = 22.349 (2) Å	*T* = 298 K
*V* = 3694.3 (6) Å^3^	Block, yellow
*Z* = 8	0.41 × 0.30 × 0.27 mm

### Data collection

**Table:**

Bruker APEX CCD diffractometer	3266 independent reflections
Radiation source: fine-focus sealed tube	2789 reflections with *I* > 2σ(*I*)
graphite	*R*_int_ = 0.045
φ and ω scans	θ_max_ = 25.0°, θ_min_ = 2.2°
Absorption correction: multi-scan (*SADABS*; Bruker, 2001)	*h* = −10→7
*T*_min_ = 0.622, *T*_max_ = 0.695	*k* = −22→22
17188 measured reflections	*l* = −22→26

### Refinement

**Table:**

Refinement on *F*^2^	Secondary atom site location: difference Fourier map
Least-squares matrix: full	Hydrogen site location: inferred from neighbouring sites
*R*[*F*^2^ > 2σ(*F*^2^)] = 0.033	H-atom parameters constrained
*wR*(*F*^2^) = 0.065	*w* = 1/[σ^2^(*F*_o_^2^) + (0.0047*P*)^2^ + 7.3452*P*] where *P* = (*F*_o_^2^ + 2*F*_c_^2^)/3
*S* = 1.16	(Δ/σ)_max_ = 0.001
3266 reflections	Δρ_max_ = 0.52 e Å^−^^3^
236 parameters	Δρ_min_ = −0.46 e Å^−^^3^
0 restraints	Extinction correction: *SHELXL97* (Sheldrick, 2008), Fc^*^=kFc[1+0.001xFc^2^λ^3^/sin(2θ)]^-1/4^
Primary atom site location: structure-invariant direct methods	Extinction coefficient: 0.00446 (16)

### Fractional atomic coordinates and isotropic or equivalent isotropic displacement parameters (Å^2^)

**Table:**

	*x*	*y*	*z*	*U*_iso_*/*U*_eq_	
Cd1	0.63465 (3)	0.587420 (13)	0.411381 (11)	0.03164 (12)	
N1	0.7363 (4)	0.52699 (16)	0.33033 (14)	0.0367 (7)	
N2	0.5467 (4)	0.64254 (17)	0.32424 (14)	0.0379 (8)	
O1	0.4794 (3)	0.49034 (13)	0.42568 (12)	0.0433 (7)	
H1A	0.5121	0.4505	0.4127	0.052*	
H1B	0.4110	0.4828	0.4516	0.052*	
O2	0.7916 (3)	0.68309 (13)	0.42330 (11)	0.0392 (6)	
H2A	0.7508	0.7241	0.4202	0.047*	
H2B	0.8638	0.6858	0.4483	0.047*	
O3	0.4697 (3)	0.64814 (14)	0.46954 (13)	0.0505 (8)	
H3A	0.4856	0.6921	0.4769	0.061*	
H3B	0.3776	0.6393	0.4775	0.061*	
O4	0.7684 (3)	0.53557 (13)	0.48847 (12)	0.0433 (7)	
H4A	0.7885	0.4911	0.4880	0.052*	
H4B	0.8429	0.5579	0.5037	0.052*	
O5	0.8325 (3)	0.31356 (13)	0.41435 (12)	0.0445 (7)	
O6	1.0491 (3)	0.38259 (15)	0.44410 (13)	0.0500 (8)	
O7	0.8156 (3)	0.39447 (14)	0.49742 (14)	0.0517 (8)	
O8	0.9605 (3)	0.28672 (13)	0.50624 (13)	0.0489 (8)	
O9	0.9479 (3)	0.86732 (14)	0.37107 (13)	0.0499 (8)	
H9A	0.8684	0.8471	0.3842	0.060*	
H9B	1.0241	0.8436	0.3830	0.060*	
O10	0.7963 (3)	0.20834 (17)	0.59585 (14)	0.0601 (8)	
H10A	0.8254	0.2339	0.5666	0.072*	
H10B	0.7303	0.1791	0.5829	0.072*	
S1	0.91417 (11)	0.34438 (4)	0.46528 (4)	0.0313 (2)	
C1	0.8259 (5)	0.4706 (2)	0.3331 (2)	0.0528 (12)	
H1	0.8577	0.4545	0.3704	0.063*	
C2	0.8748 (6)	0.4343 (3)	0.2817 (3)	0.0679 (15)	
H2	0.9362	0.3941	0.2850	0.082*	
C3	0.8315 (6)	0.4584 (3)	0.2274 (3)	0.0711 (16)	
H3	0.8640	0.4348	0.1931	0.085*	
C4	0.7386 (5)	0.5184 (3)	0.22216 (19)	0.0513 (12)	
C5	0.6928 (4)	0.5516 (2)	0.27550 (16)	0.0370 (9)	
C6	0.5959 (4)	0.6128 (2)	0.27248 (16)	0.0361 (9)	
C7	0.5517 (5)	0.6411 (3)	0.21632 (18)	0.0500 (12)	
C8	0.4601 (6)	0.7016 (3)	0.2162 (2)	0.0628 (14)	
H8	0.4307	0.7218	0.1800	0.075*	
C9	0.4140 (6)	0.7310 (3)	0.2677 (2)	0.0643 (14)	
H9	0.3534	0.7716	0.2676	0.077*	
C10	0.4585 (5)	0.6996 (2)	0.3217 (2)	0.0508 (11)	
H10	0.4246	0.7197	0.3574	0.061*	
C11	0.6927 (6)	0.5484 (3)	0.1662 (2)	0.0679 (15)	
H11	0.7245	0.5269	0.1308	0.081*	
C12	0.6042 (6)	0.6069 (3)	0.1636 (2)	0.0663 (15)	
H12	0.5770	0.6254	0.1265	0.080*	

### Atomic displacement parameters (Å^2^)

**Table:**

	*U*^11^	*U*^22^	*U*^33^	*U*^12^	*U*^13^	*U*^23^
Cd1	0.03948 (18)	0.02569 (15)	0.02976 (16)	0.00082 (12)	0.00038 (12)	−0.00058 (12)
N1	0.0370 (18)	0.0301 (17)	0.0429 (19)	−0.0027 (15)	0.0088 (15)	−0.0022 (14)
N2	0.040 (2)	0.0347 (18)	0.0389 (18)	−0.0004 (15)	−0.0040 (16)	0.0039 (14)
O1	0.0472 (17)	0.0317 (14)	0.0510 (17)	−0.0061 (13)	0.0075 (14)	−0.0014 (12)
O2	0.0394 (15)	0.0253 (13)	0.0530 (17)	0.0024 (12)	−0.0097 (13)	−0.0017 (12)
O3	0.0479 (18)	0.0342 (15)	0.069 (2)	−0.0064 (13)	0.0238 (15)	−0.0175 (14)
O4	0.0484 (17)	0.0236 (13)	0.0579 (17)	0.0007 (12)	−0.0196 (14)	0.0005 (12)
O5	0.0478 (17)	0.0312 (14)	0.0545 (17)	−0.0085 (13)	−0.0188 (14)	0.0023 (13)
O6	0.0409 (17)	0.0461 (17)	0.0632 (19)	−0.0136 (14)	0.0002 (15)	−0.0023 (14)
O7	0.0480 (18)	0.0301 (15)	0.077 (2)	0.0033 (13)	0.0094 (16)	−0.0063 (14)
O8	0.0584 (19)	0.0302 (15)	0.0580 (18)	0.0041 (13)	−0.0206 (15)	0.0032 (13)
O9	0.0431 (17)	0.0403 (16)	0.066 (2)	0.0016 (14)	0.0109 (15)	0.0069 (14)
O10	0.0499 (19)	0.061 (2)	0.070 (2)	0.0017 (16)	−0.0032 (17)	−0.0018 (16)
S1	0.0291 (5)	0.0196 (4)	0.0451 (5)	−0.0003 (4)	−0.0060 (4)	−0.0012 (4)
C1	0.050 (3)	0.041 (2)	0.067 (3)	0.003 (2)	0.015 (2)	0.000 (2)
C2	0.062 (3)	0.052 (3)	0.090 (4)	0.009 (3)	0.026 (3)	−0.018 (3)
C3	0.067 (4)	0.077 (4)	0.069 (4)	−0.006 (3)	0.026 (3)	−0.027 (3)
C4	0.046 (3)	0.062 (3)	0.046 (3)	−0.015 (2)	0.016 (2)	−0.013 (2)
C5	0.032 (2)	0.045 (2)	0.035 (2)	−0.0137 (18)	0.0064 (17)	−0.0060 (17)
C6	0.032 (2)	0.043 (2)	0.034 (2)	−0.0137 (18)	0.0001 (17)	0.0022 (17)
C7	0.044 (3)	0.068 (3)	0.038 (2)	−0.023 (2)	−0.004 (2)	0.007 (2)
C8	0.056 (3)	0.073 (3)	0.060 (3)	−0.014 (3)	−0.019 (3)	0.026 (3)
C9	0.061 (3)	0.058 (3)	0.074 (4)	−0.003 (3)	−0.020 (3)	0.015 (3)
C10	0.051 (3)	0.045 (3)	0.056 (3)	0.003 (2)	−0.008 (2)	0.004 (2)
C11	0.066 (3)	0.101 (4)	0.036 (3)	−0.019 (3)	0.013 (2)	−0.013 (3)
C12	0.062 (3)	0.101 (4)	0.036 (3)	−0.020 (3)	−0.001 (2)	0.007 (3)

### Geometric parameters (Å, °)

**Table:**

Cd1—O3	2.260 (3)	O9—H9B	0.8500
Cd1—O2	2.280 (2)	O10—H10A	0.8500
Cd1—O1	2.298 (3)	O10—H10B	0.8500
Cd1—O4	2.304 (2)	C1—C2	1.403 (6)
Cd1—N1	2.317 (3)	C1—H1	0.9300
Cd1—N2	2.337 (3)	C2—C3	1.350 (7)
N1—C1	1.320 (5)	C2—H2	0.9300
N1—C5	1.364 (5)	C3—C4	1.396 (7)
N2—C10	1.323 (5)	C3—H3	0.9300
N2—C6	1.355 (5)	C4—C5	1.403 (5)
O1—H1A	0.8500	C4—C11	1.429 (7)
O1—H1B	0.8499	C5—C6	1.432 (6)
O2—H2A	0.8499	C6—C7	1.417 (5)
O2—H2B	0.8500	C7—C8	1.392 (7)
O3—H3A	0.8500	C7—C12	1.418 (7)
O3—H3B	0.8500	C8—C9	1.340 (7)
O4—H4A	0.8500	C8—H8	0.9300
O4—H4B	0.8500	C9—C10	1.398 (6)
O5—S1	1.466 (3)	C9—H9	0.9300
O6—S1	1.469 (3)	C10—H10	0.9300
O7—S1	1.467 (3)	C11—C12	1.347 (7)
O8—S1	1.473 (3)	C11—H11	0.9300
O9—H9A	0.8500	C12—H12	0.9300
			
O3—Cd1—O2	86.06 (9)	O7—S1—O8	109.16 (18)
O3—Cd1—O1	86.08 (10)	O6—S1—O8	109.31 (18)
O2—Cd1—O1	165.28 (9)	N1—C1—C2	122.2 (5)
O3—Cd1—O4	96.46 (11)	N1—C1—H1	118.9
O2—Cd1—O4	86.02 (9)	C2—C1—H1	118.9
O1—Cd1—O4	82.52 (9)	C3—C2—C1	119.1 (5)
O3—Cd1—N1	160.98 (12)	C3—C2—H2	120.4
O2—Cd1—N1	103.77 (10)	C1—C2—H2	120.4
O1—Cd1—N1	87.42 (10)	C2—C3—C4	120.8 (5)
O4—Cd1—N1	100.39 (10)	C2—C3—H3	119.6
O3—Cd1—N2	92.46 (11)	C4—C3—H3	119.6
O2—Cd1—N2	87.35 (10)	C3—C4—C5	116.9 (4)
O1—Cd1—N2	105.41 (10)	C3—C4—C11	123.8 (5)
O4—Cd1—N2	168.48 (11)	C5—C4—C11	119.3 (5)
N1—Cd1—N2	72.09 (11)	N1—C5—C4	122.2 (4)
C1—N1—C5	118.7 (4)	N1—C5—C6	118.7 (3)
C1—N1—Cd1	125.8 (3)	C4—C5—C6	119.0 (4)
C5—N1—Cd1	115.4 (2)	N2—C6—C7	120.9 (4)
C10—N2—C6	119.0 (3)	N2—C6—C5	118.7 (3)
C10—N2—Cd1	126.0 (3)	C7—C6—C5	120.4 (4)
C6—N2—Cd1	115.0 (2)	C8—C7—C6	117.8 (4)
Cd1—O1—H1A	116.2	C8—C7—C12	123.7 (5)
Cd1—O1—H1B	130.6	C6—C7—C12	118.5 (5)
H1A—O1—H1B	109.3	C9—C8—C7	120.5 (4)
Cd1—O2—H2A	116.1	C9—C8—H8	119.7
Cd1—O2—H2B	125.5	C7—C8—H8	119.7
H2A—O2—H2B	108.6	C8—C9—C10	119.0 (5)
Cd1—O3—H3A	119.4	C8—C9—H9	120.5
Cd1—O3—H3B	129.9	C10—C9—H9	120.5
H3A—O3—H3B	107.8	N2—C10—C9	122.7 (5)
Cd1—O4—H4A	120.7	N2—C10—H10	118.6
Cd1—O4—H4B	119.3	C9—C10—H10	118.6
H4A—O4—H4B	108.8	C12—C11—C4	121.4 (5)
H9A—O9—H9B	108.3	C12—C11—H11	119.3
H10A—O10—H10B	107.9	C4—C11—H11	119.3
O5—S1—O7	109.79 (17)	C11—C12—C7	121.3 (5)
O5—S1—O6	109.95 (17)	C11—C12—H12	119.3
O7—S1—O6	109.24 (17)	C7—C12—H12	119.3
O5—S1—O8	109.38 (15)		
			
O3—Cd1—N1—C1	−141.9 (4)	Cd1—N1—C5—C6	2.5 (4)
O2—Cd1—N1—C1	98.5 (3)	C3—C4—C5—N1	0.1 (6)
O1—Cd1—N1—C1	−71.8 (3)	C11—C4—C5—N1	−178.0 (4)
O4—Cd1—N1—C1	10.1 (3)	C3—C4—C5—C6	−179.2 (4)
N2—Cd1—N1—C1	−178.9 (3)	C11—C4—C5—C6	2.7 (6)
O3—Cd1—N1—C5	35.5 (5)	C10—N2—C6—C7	−1.2 (6)
O2—Cd1—N1—C5	−84.1 (3)	Cd1—N2—C6—C7	−179.7 (3)
O1—Cd1—N1—C5	105.6 (3)	C10—N2—C6—C5	179.2 (3)
O4—Cd1—N1—C5	−172.5 (2)	Cd1—N2—C6—C5	0.7 (4)
N2—Cd1—N1—C5	−1.5 (2)	N1—C5—C6—N2	−2.2 (5)
O3—Cd1—N2—C10	13.4 (3)	C4—C5—C6—N2	177.1 (4)
O2—Cd1—N2—C10	−72.6 (3)	N1—C5—C6—C7	178.2 (3)
O1—Cd1—N2—C10	100.0 (3)	C4—C5—C6—C7	−2.5 (5)
O4—Cd1—N2—C10	−127.4 (5)	N2—C6—C7—C8	1.9 (6)
N1—Cd1—N2—C10	−178.0 (4)	C5—C6—C7—C8	−178.6 (4)
O3—Cd1—N2—C6	−168.3 (3)	N2—C6—C7—C12	−178.8 (4)
O2—Cd1—N2—C6	105.8 (3)	C5—C6—C7—C12	0.7 (6)
O1—Cd1—N2—C6	−81.6 (3)	C6—C7—C8—C9	−1.0 (7)
O4—Cd1—N2—C6	50.9 (6)	C12—C7—C8—C9	179.7 (5)
N1—Cd1—N2—C6	0.4 (2)	C7—C8—C9—C10	−0.4 (7)
C5—N1—C1—C2	−1.6 (6)	C6—N2—C10—C9	−0.3 (6)
Cd1—N1—C1—C2	175.7 (3)	Cd1—N2—C10—C9	178.0 (3)
N1—C1—C2—C3	1.4 (8)	C8—C9—C10—N2	1.2 (7)
C1—C2—C3—C4	−0.4 (8)	C3—C4—C11—C12	−179.2 (5)
C2—C3—C4—C5	−0.3 (7)	C5—C4—C11—C12	−1.2 (7)
C2—C3—C4—C11	177.8 (5)	C4—C11—C12—C7	−0.6 (8)
C1—N1—C5—C4	0.8 (6)	C8—C7—C12—C11	−180.0 (5)
Cd1—N1—C5—C4	−176.8 (3)	C6—C7—C12—C11	0.8 (7)
C1—N1—C5—C6	−179.9 (3)		

### Hydrogen-bond geometry (Å, °)

**Table:**

*D*—H···*A*	*D*—H	H···*A*	*D*···*A*	*D*—H···*A*
O1—H1A···O9^i^	0.85	1.85	2.682 (4)	167
O1—H1B···O4^ii^	0.85	2.10	2.952 (4)	176
O2—H2A···O5^iii^	0.85	1.83	2.682 (3)	178
O2—H2B···O8^iv^	0.85	1.93	2.757 (4)	165
O3—H3A···O8^iii^	0.85	1.95	2.787 (4)	170
O3—H3B···O7^ii^	0.85	1.90	2.746 (4)	170
O4—H4A···O7	0.85	1.84	2.679 (4)	171
O4—H4B···O6^iv^	0.85	1.87	2.686 (4)	159
O9—H9A···O5^iii^	0.85	2.00	2.844 (4)	172
O9—H9B···O10^iv^	0.85	1.92	2.768 (4)	176
O10—H10A···O8	0.85	2.05	2.875 (4)	162
O10—H10B···O6^v^	0.85	2.06	2.909 (4)	172

Symmetry codes: (i) −*x*+3/2, *y*−1/2, *z*; (ii) −*x*+1, −*y*+1, −*z*+1; (iii) −*x*+3/2, *y*+1/2, *z*; (iv) −*x*+2, −*y*+1, −*z*+1; (v) *x*−1/2, −*y*+1/2, −*z*+1.
